# Modulation of inflammasome components in patients with heart failure using oral nutritional supplements: investigating the molecular mechanisms beyond the clinical benefit

**DOI:** 10.1007/s00394-025-03878-5

**Published:** 2026-02-03

**Authors:** Aura D. Herrera-Martínez, Natalia Hermán-Sánchez, Miguel E. G-García, Concepción Muñoz-Jiménez, Jesús M. Pérez-Gómez, Antonio J. Montero-Hidalgo, José López-Aguilera, Rafael González-Manzanares, María Ángeles Gálvez-Moreno, María José Molina-Puerta, Raúl M. Luque

**Affiliations:** 1 Maimonides Institute for Biomedical Research of Cordoba (IMIBIC), Edificio IMIBIC. Av. Menéndez Pidal s/n, 14004 Córdoba, Spain; 2 Endocrinology and Nutrition Service, Reina Sofia University Hospital, Córdoba, Spain; 3 Department of Cell Biology, Physiology, and Immunology, University of Córdoba, Córdoba, Spain; 4 Cardiology Service, Reina Sofía University Hospital, Córdoba, Spain; 5 CIBER Enfermedades Cardiovasculares, Madrid, Spain; 6 CIBER Fisiopatología de la Obesidad y Nutrición (CIBERobn), Madrid, Spain

**Keywords:** Oral supplements, Heart failure, Inflammasome, Heart remodeling, Outcomes

## Abstract

**Purpose:**

Inflammation is a key contributor to the pathogenesis and progression of heart failure (HF), correlating with increased morbidity and mortality. This study aimed to evaluate the molecular impact of a 24-week nutritional intervention on inflammasome-related components in HF patients, comparing a Mediterranean diet alone versus the same diet supplemented with hypercaloric, high-protein oral nutritional supplements (ONS) enriched with eicosapentaenoic acid (EPA) and docosahexaenoic acid (DHA). In a cohort of 38 patients, expression levels of inflammasome markers were assessed via microfluidic quantitative polymerase chain reaction (PCR) in peripheral blood mononuclear cells at baseline and post-intervention.

**Results:**

Some components, especially cytokines and apoptosis regulation components are overexpressed in patients with sarcopenia (NLRP1, NLRC4, CASP1, CASP5, CTSL, IFI16, TLR8, PSXR7, CCR1, CHUCK, MAPK14, CDKN1B). We observed a significant downregulation of Nod-like receptors NLRP12 and NLRP6, along with decreased expression of inflammasome activation components CASP5, TLR2, and TLR9 in the intervention group (*p* < 0.05). Additionally, cytokines and inflammation-related molecules such as CXCR1, CXCR2, TGFB, CCL2, and NF-κB showed reduced expression, while the inhibitor CHUCK increased (*p* < 0.05). Cell cycle regulators also shifted, with decreased CDKN2D expression (*p* < 0.05), suggesting potential effects on cellular senescence and DNA repair pathways. Notably, these molecular changes were absent in patients adhering solely to the Mediterranean diet.

**Conclusions:**

these findings suggest that supplementing a Mediterranean diet with hypercaloric, high-protein, EPA and DHA-enriched ONS induces molecular modifications in inflammasome pathways associated with cardiac remodeling. Therefore, targeted nutritional strategies may offer a promising adjunct to improve cardiac function and disease progression in HF patients.

**Supplementary Information:**

The online version contains supplementary material available at 10.1007/s00394-025-03878-5.

## Introduction

Patients with heart failure (HF) often present with significant cardiac and non-cardiac comorbidities [1–3], which are associated with a more adverse clinical course and increased mortality rates [4, 5]. Among the various pathogenic mechanisms, inflammation plays a pivotal role in the development and progression of HF. Elevated levels of proinflammatory cytokines are commonly observed in patients with HF and correlate with the severity of cardiac dysfunction. Furthermore, systemic inflammation has been linked to poorer clinical outcomes and serves as an independent prognostic factor for left ventricular ejection fraction (LVEF) [6, 7].

Inflammasomes are large, multiprotein cytoplasmic complexes composed of a sensor protein, inflammatory caspases, and, in most cases, an adaptor protein that functions as an intracellular sensor. These complexes are critical mediators of inflammatory responses within the innate immune system. Inflammasomes can be activated by a diverse array of internal and external stimuli, leading to the enzymatic activation of canonical caspases. This activation results in the processing and release of interleukins, as well as the induction of cell death pathways such as apoptosis and pyroptosis [8, 9]. Proper regulation of inflammasome activation is essential for host defense against pathogens and tissue injury; however, dysregulated or excessive activation can contribute to pathological tissue responses, playing a role in various diseases, including autoinflammatory conditions, obesity, and cardiometabolic disorders [8, 10, 11].

Inflammasome activation has been implicated in the pathogenesis of HF, contributing to maladaptive cardiac remodeling and the loss of cardiomyocytes through processes such as hypertrophy, fibrosis, and pyroptosis. Notably, activation of the NOD-like receptor protein 3 (NLRP3) inflammasome has been shown to promote myocardial hypertrophy under conditions of pressure overload [12]. The secretion of some interleukins (ILs), specifically, IL1β and IL18, promote heart fibrosis [13], while heightened NLRP3 activation induces cardiomyocyte pyroptosis, ultimately leading to myocardial dysfunction and dilated cardiomyopathy, which are key contributors to HF progression [14].

In this context, nutritional interventions have demonstrated potential in improving both cardiac function markers and circulating inflammatory mediators [6, 15]. Specifically, several studies suggest that supplementation with omega-3 fatty acids reduces circulating cytokine levels in patients with HF, indicating a possible strategy for attenuating inflammation in this population [16]. However, the precise impact of such nutritional supplementation on the molecular expression of inflammasome components remains to be elucidated. Moreover, there is currently no consensus regarding the optimal dosage or mode of administration.

Given that gene expression profiles in peripheral blood mononuclear cells (PBMCs) often reflect and mirror disease-specific molecular signatures [17]. this study hypothesizes that the expression patterns of key inflammasome components within PBMCs may be associated with cardiac remodeling and functional status in patients with HF. According to previous investigations conducted by our research group, we observed that a combined intervention comprising a Mediterranean diet, calcifediol supplementation, and nutritional support with a high-protein, hypercaloric oral nutritional supplement (ONS) enriched with omega-3 (n-3) and omega-6 (n-6) polyunsaturated fatty acids (PUFAs) resulted in significant improvements in cardiac-related biomarkers. Specifically, patients exhibited increased left ventricular ejection fraction (LVEF) and decreased serum levels of N-terminal pro-B-type natriuretic peptide (NT-proBNP) after twenty-four weeks, compared to those receiving only the Mediterranean diet with calcifediol. These improvements were also associated with increased muscle mass without concomitant body weight gain [15].

Given these notable clinical outcomes, we sought to investigate whether the underlying molecular expression of inflammasome-related genes in PBMCs might be correlated with the observed functional and structural cardiac benefits.

## Material and methods

### Patients

This study was approved by the Ethics Committee of the Reina Sofia University Hospital (Cordoba, Spain; reference number 5164 approved on October 21st, 2021 and updated on May 30th, 2023) and conducted in accordance with the Declaration of Helsinki and following national and international guidelines. Cordoba Biobank Node (through Andalusian Biobank) coordinated the collection, processing, and management of the samples used. Specifically, a prospective open label study was performed, wherein a written informed consent was signed by every individual before inclusion into the study. All patients received information and only if accepted to participate, were included. This cohort was initially studied in an open, randomized, controlled, clinical trial (ClinicalTrials.gov number: NCT05848960) [15], in which patients of both sexes, age > 18 y-old < 85 y-old, LVEF < 50% and a hospital admission due to HF in the previous 6 months were included. The trial was designed and reported in accordance with CONSORT guidelines for randomized controlled clinical studies. Participants were randomly allocated in a 1:1 ratio using a computer-generated randomization sequence, with allocation concealment maintained through sealed, opaque envelopes. Although the nature of the intervention precluded blinding of participants and investigators, outcome assessors and data analysts remained blinded to group assignment to minimize bias. All predefined outcomes, sample size calculations, eligibility criteria, and deviations from the protocol were prospectively registered before study initiation. Flow of participants—including recruitment, allocation, follow-up, and analysis—was documented following the CONSORT flow diagram structure [15]. This methodological framework ensured transparency, reproducibility, and rigorous adherence to standards for high-quality clinical research. N-terminal pro-brain natriuretic peptide (NT-proBNP) was determined at baseline and at the end of the study. LVEF was evaluated using transthoracic ultrasound. Malnutrition was defined in this cohort according to the GLIM criteria [18] and sarcopenia was defined using handgrip strength according to the updated European Working Group on Sarcopenia in older people (EWGSOP2 [19].

### Nutritional support

In this randomized clinical trial, participants were allocated in a 1:1 ratio to either a control group receiving a Mediterranean diet alone or a treatment group receiving the Mediterranean diet supplemented with two hypercaloric, high-protein oral nutritional supplements (ONS) per day, over a period of twenty-four weeks. At baseline, all patients received standardized education and guidance regarding nutritional support, adherence to the Mediterranean diet, and physical activity. Additionally, all participants were supplemented with calcifediol to achieve serum 25-hydroxyvitamin D levels exceeding 30 ng/mL.

The ONS provided consisted of 200 mL of a hypercaloric (141 kcal/100 mL), high-protein (7.4 g/100 mL) nutritional formula. This formulation also included slow-release carbohydrates (14.5 g/100 mL with 0.86 g/100 mL of sugars), a fiber mixture (1.7 g/100 mL), and a combination of omega-3 and omega-6 fatty acids, specifically eicosapentaenoic acid (EPA) and docosahexaenoic acid (DHA) at a concentration of 385 mg/100 mL. The supplements were kindly provided by Vegenat Healthcare®.

Inclusion criteria mandated that only patients with a treatment adherence of at least 75% be considered for analysis. Nineteen patients were initially enrolled in each arm; however, due to mortality, the final analysis included fifteen patients in the control group and eighteen in the treatment group.

### Blood sampling and processing to isolate PBMCs

Venous blood from all patients was collected in EDTA-coated tubes at baseline and at the end of the study. *Human peripheral blood mononuclear cells (PBMCs*) were isolated as previously described [17].

### Total RNA isolation and retrotranscription

Total RNA from PBMCs was isolated using the Direct-zol RNA kit (Zymo Research, Irvine, CA, USA) following manufacturer's instructions. Following extraction, RNA samples underwent DNase treatment using the AllPrep DNA/RNA/Protein Kit (QIAGEN) to eliminate any contaminating genomic DNA rest. The amount of RNA recovered was determined and its quality assessed by the NanoDrop2000 spectrophotometer (Thermo Fisher). All the RNA samples passed the quality controls, with 260/280 and 260/230 absorbance ratios ranging between 1.8 and 2.0. One μg of RNA was reverse transcribed (RT) into cDNA using random hexamer primers with the First Strand Synthesis Kit (Thermo Fisher), as previously described [10]. The resulting cDNA was stored at − 20 °C until further use.

### Analysis of inflammasome components by quantitative polymerase chain reaction (qPCR) dynamic array based on microfluidic technology

As previously described a microfluidic-based qPCR array (Standard BioTools, San Francisco, CA, USA) was used to simultaneously determine the gene expression levels of 48 candidate genes belonging to the inflammasome complex in all patients using a 96.96 Dynamic Array Plate (GE 48.48 Dynamic Array Reagent Kit with Control Line Fluid, Standard BioTools, San Francisco, CA, USA). Briefly, specific primers for human transcripts including NLR-/NOD-like receptors, regulators of inflammasome activation, cytokines and inflammation/apoptosis-related components, cell-cycle and DNA-damage regulators were used as previously reported [10, 17, 20, 21]. The expression level of each transcript was adjusted by a normalization factor obtained from the mRNA levels of 3 different housekeeping genes [Beta actin (ACTB), Glyceraldehyde-3-Phosphate Dehydrogenase (GAPDH) and *Hypoxanthine Phosphoribosyltransferase 1* (HPRT)] using Genorm 3.3 [22]. These genes were selected based on their expression stability across the experimental groups, as none showed significant variation between groups (data not shown).

Preamplification, exonuclease I treatment, and microfluidic-based qPCR array were implemented as previously described [17, 20, 21] following the manufacturer's instructions, using the Biomark HD System and the Fluidigm Real-Time PCR Analysis Software.

### Statistical analysis

Statistical analyses were performed using SPSS statistical software version 20, and Graph Pad Prism version 10. Between-group comparisons were analyzed by the Mann–Whitney U test (nonparametric data). Within-subject comparisons between baseline and 24 weeks were performed using the Wilcoxon signed-rank test. To correct for multiple testing across variables, *p*-values were adjusted using the Holm–Bonferroni method. Statistical significance was defined as adjusted *p* < 0.05. The chi-squared test was used to compare categorical data. Data are expressed as median with interquartile range and percentages. Absolute differences in some parameters were calculated using mean values. For specific group analysis, the absolute number has also been expressed in brackets. *P*-values < 0.05 were considered statistically significant.

## Results

### Baseline characteristics of the groups

The general characteristics of the included patients are summarized in Supplementary Table 1. In brief, a total of thirty-eight patients were enrolled, with a mean age of 66.7 years. The cohort was predominantly male, comprising 71.1% (*n* = 27) of the participants. According to the GLIM criteria, 57.9% (*n* = 22) of patients were classified as malnourished; however, sarcopenia was specifically identified in 28.9% (*n* = 11) of the cohort.

In the whole cohort, the mean baseline left ventricular ejection fraction (LVEF) was 38.5%. During the previous hospital admission, patients exhibited a mean serum NT-proBNP level of 5,768 pg/mL. Following 24 weeks of nutritional intervention, a significant reduction in NT-proBNP levels was observed, decreasing from 3225 ± 3882 pg/mL to 1300 ± 1226 pg/mL (*p* < 0.01). Regarding inflammation-related biomarkers, ferritin levels showed a significant decline from a mean of 124.0 ± 99.2 mg/dL to 86.1 ± 77.4 mg/dL (*p* = 0.003). Similarly, C-reactive protein (CRP) levels decreased from 8.5 ± 15.0 mg/L to 2.8 ± 4.8 mg/L following the nutritional intervention (*p* = 0.02).

### Molecular expression of inflammasome components in heart failure patients

A total of forty-eight genes were evaluated in this study, encompassing NLR-/NOD-like receptors (*n* = 6), regulators of inflammasome activation (*n* = 19), cytokines and components related to inflammation and apoptosis (*n* = 18), as well as cell-cycle and DNA damage regulators (*n* = 5). In this section, only those genes demonstrating statistically significant alterations are reported. The complete list of evaluated genes along with their corresponding primer sequences is provided in Supplementary Table 2.

#### Inflammasome components expression in relation to malnutrition

**Fig. 1:**
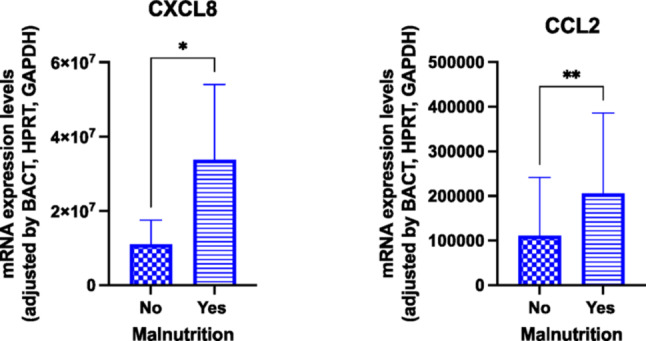
Molecular expression of inflammasome components at baseline according to the presence of malnutrition. Legend: CXCL8: chemokine CXCL8; CCL2: chemokine 2; *p < 0.05

When patients were stratified based on the presence of malnutrition, an upregulation of mRNA expression levels of the chemokines CXCL8 and CCL2 was observed in malnourished individuals compared to well-nourished counterparts (Fig. 1).

#### Inflammasome components expression in relation to sarcopenia

**Fig. 2:**
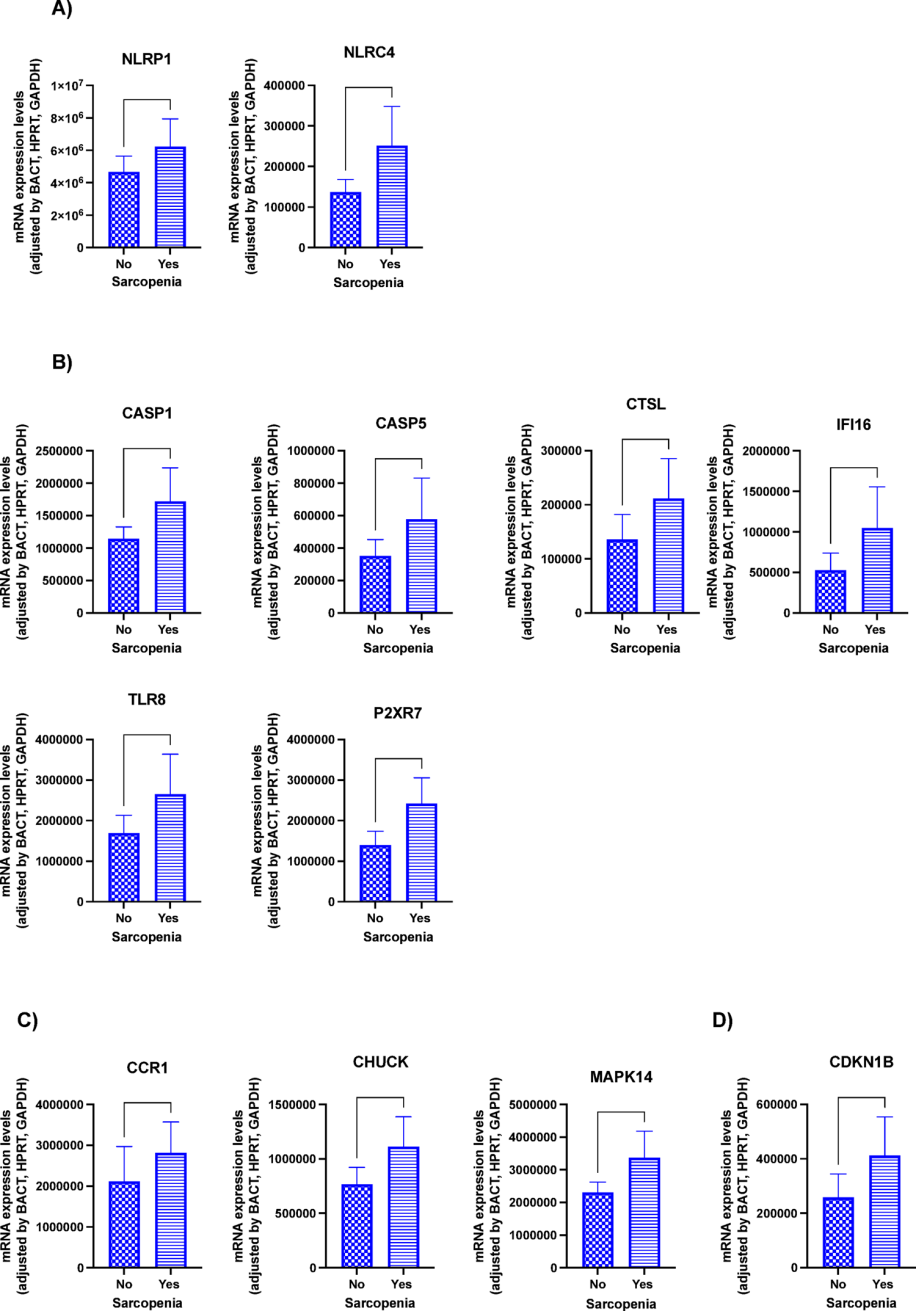
Molecular expression of inflammasome components at baseline according to the presence of sarcopenia. **A** NOD-like receptors; **B** inflammasome activation components; **C** cytokines and apoptosis related components; **D** cell cycle and DNA-damage regulators. Legend: *p < 0.05; **p < 0.01

Conversely, stratification according to sarcopenia revealed notable differences in the molecular expression profiles of these components. Specifically, NLRP1 and NLRC4 exhibited increased expression in patients with sarcopenia (Fig. 2A). This was paralleled by elevated levels of inflammasome activation components, including caspases 1 (CASP1) and 5 (CASP5), cathepsin L (CTSL), interferon gamma-inducible protein 16 (IFI16), Toll-like receptor 8 (TLR8), and the purinergic P2X receptor 7 (P2X7R) (Fig. 2B). Additionally, the expression of cytokines and apoptosis-related components such as the C–C chemokine receptor type 1 (CCR1), nuclear factor kappa B kinase complex (CHUCK), and mitogen-activated protein kinase 14 (MAPK14) was significantly increased (Fig. 2C). Similarly, the cell-cycle and DNA damage regulator cyclin dependent kinase inhibitor 1B (CDKN1B) also demonstrated elevated expression levels in patients with sarcopenia (Fig. 2D).

### Molecular changes of inflammasome components after nutritional support

As previously, only significant changes are reported in this section.

#### NOD-like receptors

**Fig. 3:**
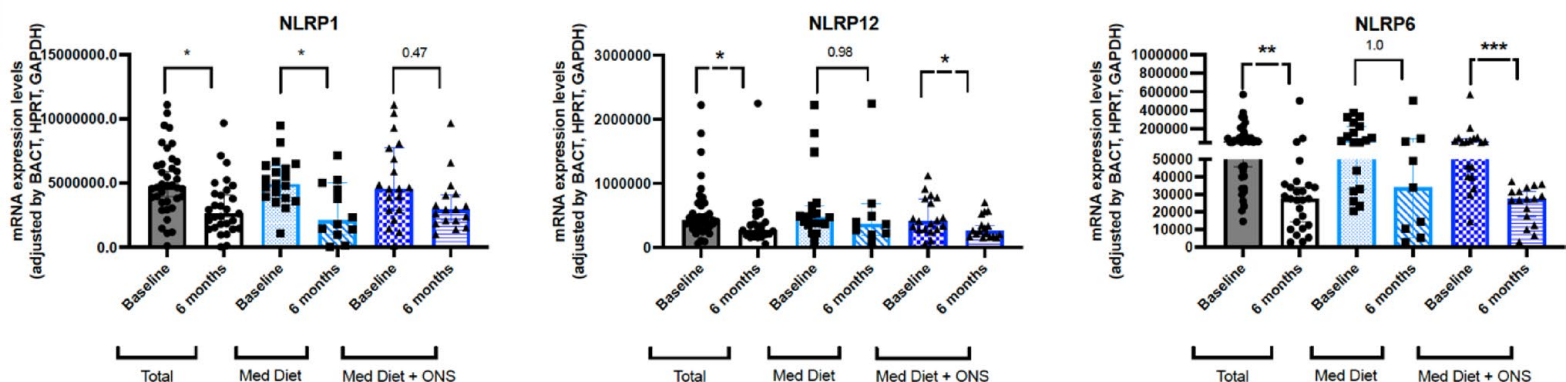
Molecular changes in the expression of NOD-like receptors in the control and intervention group. Legend: NLRP: nod-like receptor; *p < 0.05; **p < 0.01;***p < 0.001. *p*-values were adjusted using the Holm–Bonferroni method

Expression levels of NLRP1 significantly decreased in all patients after 24 weeks of intervention, being this reduction significantly higher in the control group (*p* < 0.05) compared with the intervention group (*p* = 0.47) (Fig. 3). An overall decreased in the expression levels of NLRP12 and NLR6 was observed in all patients (*p* < 0.05 and *p* < 0.01 respectively), being this change statistically significant only in the intervention group (*p* < 0.05 and *p* < 0.001 respectively), but not in the control group (*p* > 0.05) (Fig. 3). We did not observe significant changes in the molecular expression of NLRP3, NLRC4 and NLRP7 (data not shown).

#### Inflammasome activation components

**Fig. 4:**
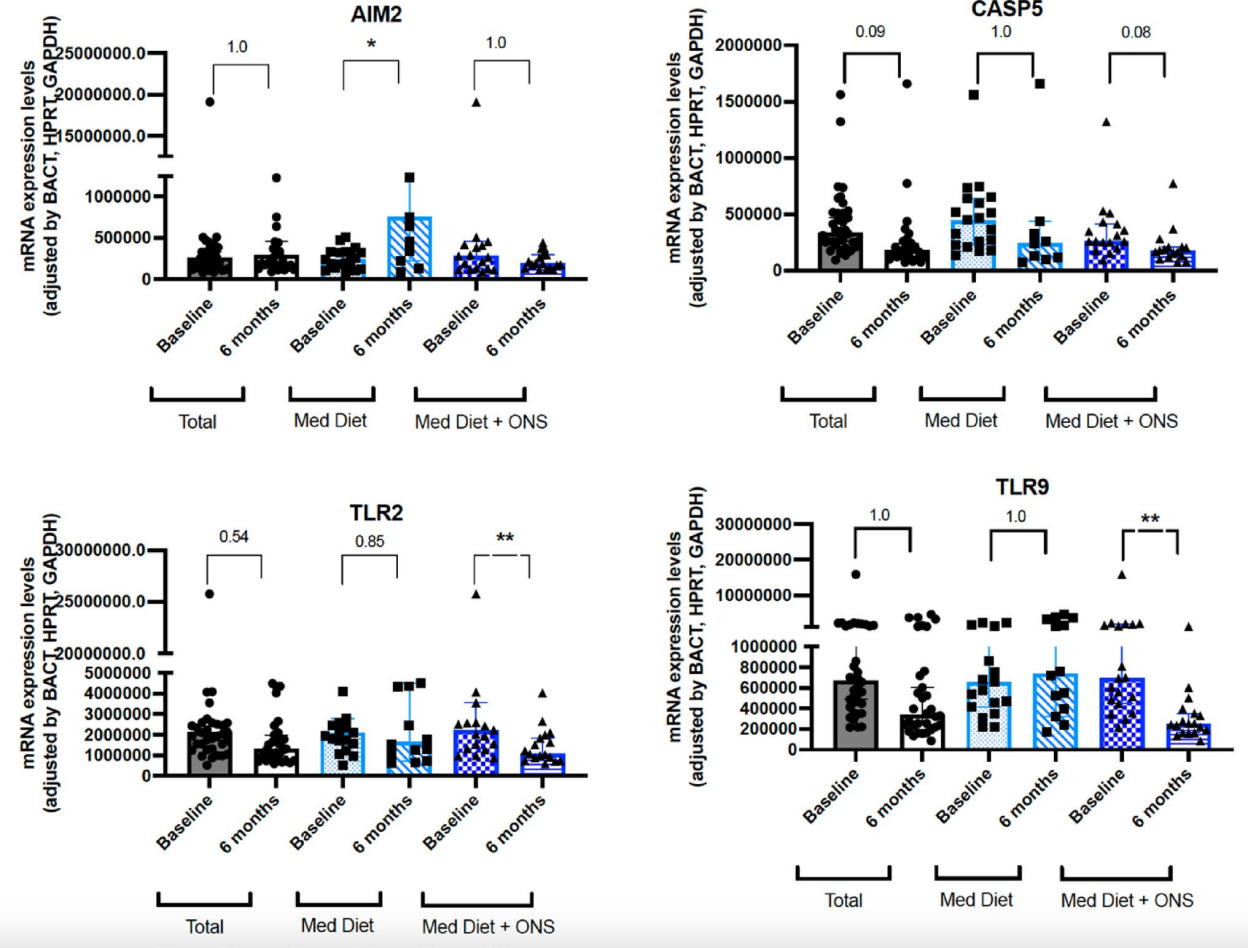
Molecular changes in the expression of the inflammasome activation components in the control and intervention group. Legend: AIM2: inflammasome protein absent in melanoma; CASP: caspase; TLR: Toll-like receptor; *p < 0.05; **p < 0.01. *p*-values were adjusted using the Holm–Bonferroni method

Remarkably, the expression of inflammasome protein absent in melanoma (AIM2), significantly increased in the control group (*p* < 0.05), while decreased in a non-significant manner in the intervention group (Fig. 4).

In general, the molecular expression of caspase 5 (CASP5) tended to decreased in all the cohort, especially in the intervention group (*p* = 0.08) and not in the control group (*p* = ns).

Similarly, TLR9 and TLR2 significantly decreased in the intervention group (*p* < 0.01) while no significant changes were observed in the control group (Fig. 4). We did not observe significant changes in the molecular expression of caspases 2, 4, 8, 9, gamma interferon (IFN-G), interleukin 1B, interleukin 18, toll-like receptor 4 (TLR4), TRL1, TLR8, PSX7 receptor, Interferon Gamma Inducible Protein 16 (IFI 16), the innate immune mediator MYD88, and gasdermin D (GDSMD) (data not shown).

#### Cytokines and inflammation/apoptosis related components

**Fig. 5:**
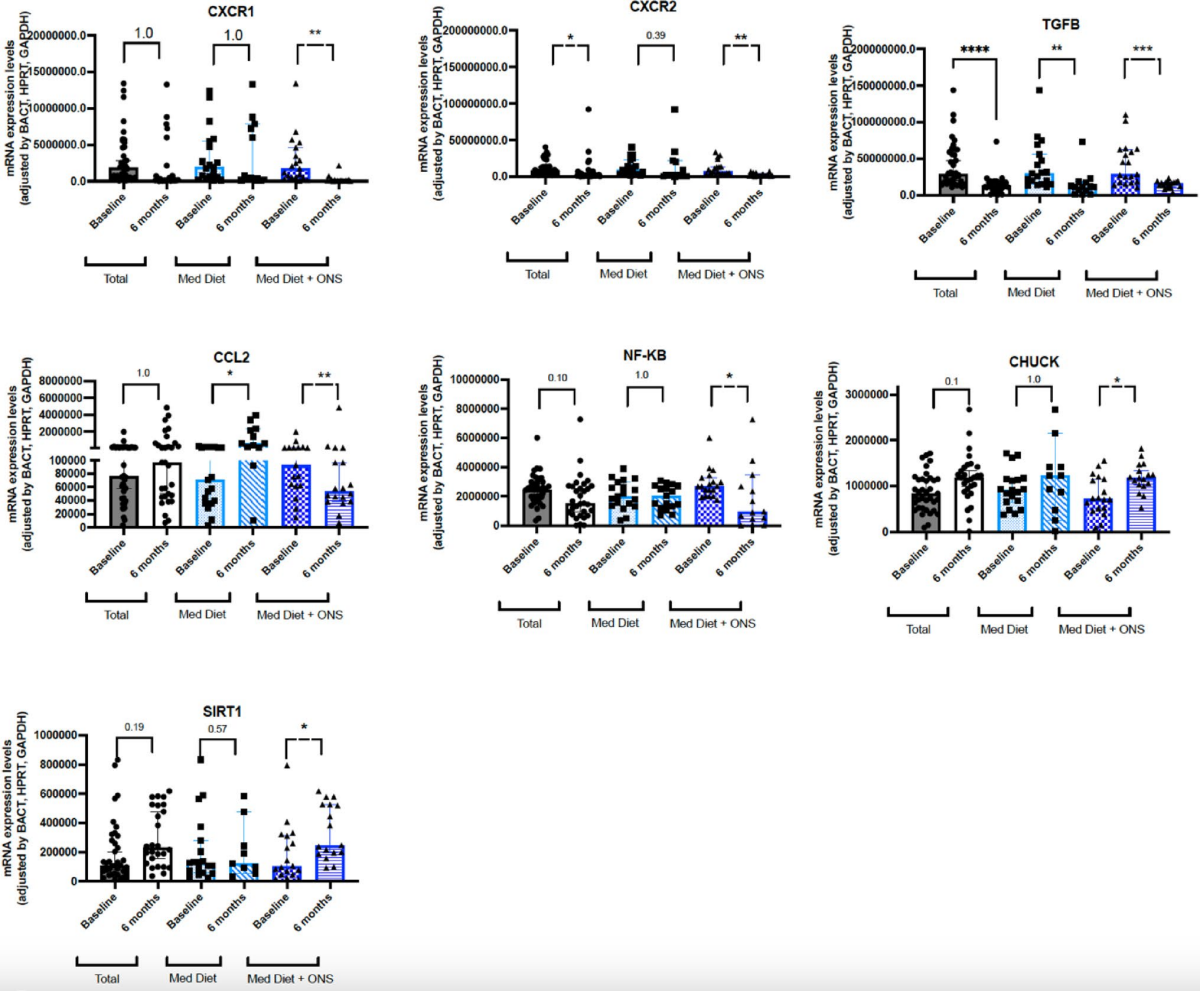
Molecular changes in the expression of cytokines in the control and intervention group. Legend: TGFB: transforming growth factor beta; NF-κB: NF-kappa-B complex; CHUK: nuclear factor kappa B kinase complex; SIRT1: sirtuin 1; IL: interleukin; *p < 0.05; **p < 0.01; ***p < 0.001; ****p < 0.0001. *p*-values were adjusted using the Holm–Bonferroni method

The expression levels of the chemokine receptor CXCR2 significantly decreased in the whole cohort (*p* < 0.05), in contrast, the decreased expression of CXXR1 and CXCR2 was only statistically relevant in the intervention group (*p* < 0.01) (Fig. 5). When the transforming growth factor beta (TGFB) was analyzed, its molecular expression significantly decreased in all patients, being this change statistically significant in both, the control (*p* < 0.01) and the intervention group (*p* < 0.001). Interestingly, while the molecular expression of CCL2 significantly increased in the control group (*p* < 0.05), it significantly decreased in the intervention group (*p* < 0.01).

The expression of the transcription factor kappa B kinase complex (NF-κB) significantly decreased in the intervention group (*p* < 0.05). In parallel, the molecular expression of CHUCK was increased in the intervention group (*p* < 0.05) but not in the control group. Similarly, the expression of sirtuin 1 (SIRT1) significantly increased in the intervention group (*p* < 0.05) but not in the control group (*p* > 0.05).

We did not observe significant changes in the molecular expression of the chemokine receptors CCR1 and CCR5, interleukin 6, interleukin 6 receptor and MAPK14 (data not shown).

#### Cell-cycle and DNA-damage regulators

**Fig. 6:**
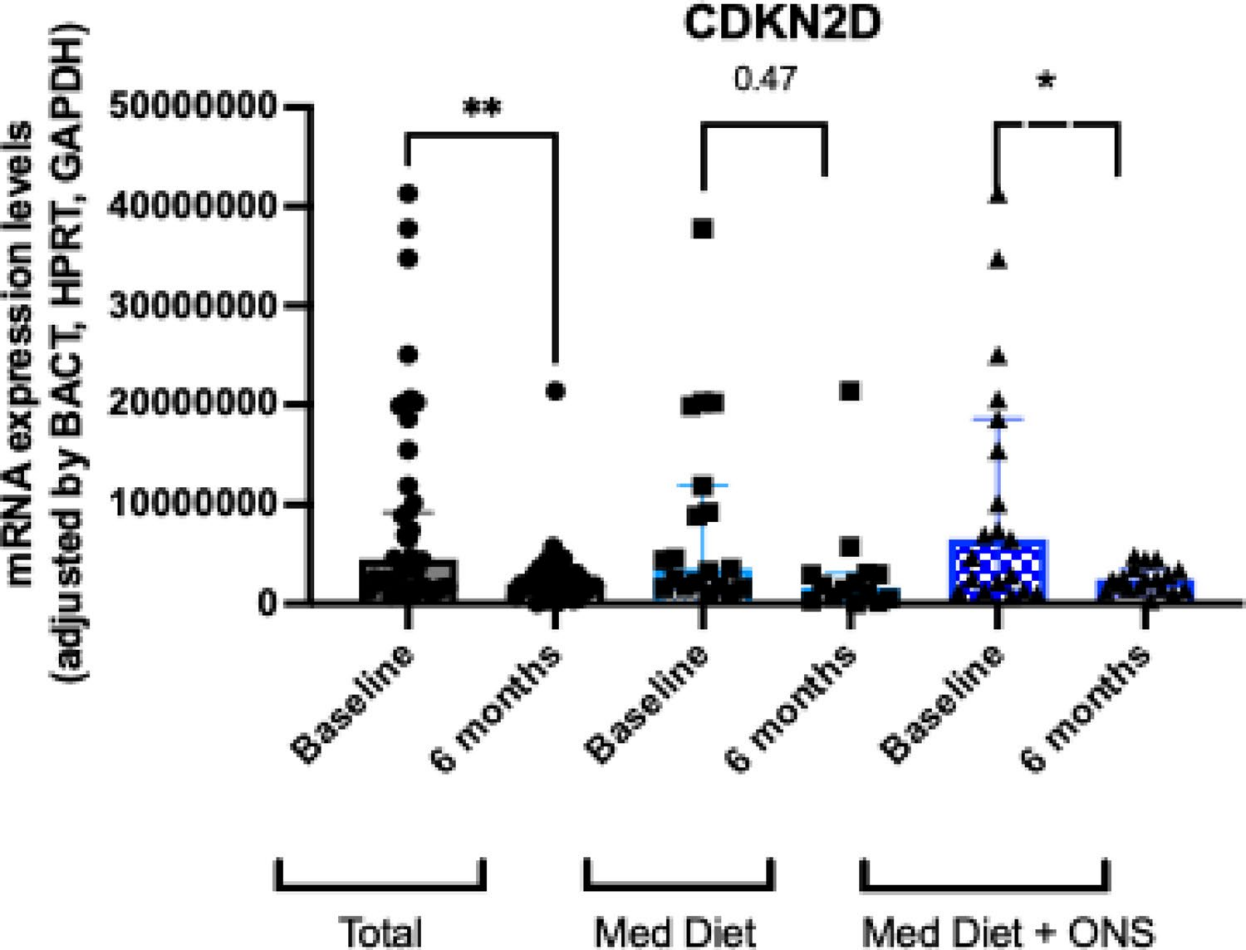
Molecular changes in the expression of cell-cycle and DNA-damage regulators in the control and intervention group. Legend: CDKN: cyclin dependent kinase inhibitor; ns: non-significant; *p < 0.05; **p < 0.01. *p*-values were adjusted using the Holm–Bonferroni method

The molecular expression of the cyclin dependent kinase inhibitor 2D (CDKN2D) significantly decreased after 24 weeks of intervention only in patients who received ONS (*p* < 0.05; Fig. 6). We did not observe significant changes in the molecular expression of CDKN1A, CDKN2A or CDKN1B (data not shown).

### Clinical association between HF-related outcomes and molecular expression of inflammasome components

An age- and sex-adjusted multivariate analysis revealed that none of the inflammasome components were independently associated with mortality within this cohort. Furthermore, the percentage changes in LVEF and NT-proBNP levels did not demonstrate a significant correlation with the percentage variations in the expression levels of the evaluated inflammasome-related components.

## Discussion

Atherosclerosis, myocardial ischemia, and HF are intricately linked to inflammatory responses and alterations, which also play a pivotal role in myocardial remodeling [23]. Moreover, recurrent and sustained activation of the immune system has been implicated in the development of left ventricular hypertrophy and its progression to HF. Previous studies have demonstrated that elevated inflammatory markers correlate with the severity and prognosis of HF [24]. Inflammation can increase the rate of cardiomyocyte apoptosis, promote cardiac hypertrophy, and activate matrix metalloproteinases, thereby potentially impairing overall cardiac function [25]. A prior investigation within this cohort revealed a significant association between nutritional support and modulation of some circulating cytokines [6]. Consequently, our objective was to determine whether the expression patterns of key inflammasome components in PBMCs of a well-characterized cohort of HF patients—who participated in a clinical trial of nutritional support—are associated with their nutritional status. Additionally, we aimed to assess whether modulation of these components, throughout nutritional support, could improve cardiac function and clinical outcomes.

Previous research has indicated that the expression of certain inflammasome components is dysregulated in HF, with some being linked to hypertrophy and fibrosis, affecting the clinical outcome of these patients [25]. Therefore, targeting myocardial remodeling through anti-inflammatory strategies appears to be a promising approach for preventing or treating HF [26]. In this context, nutritional support may play a role in modulating inflammatory processes [6, 15], this is an interesting, cost-effective intervention that has a relevant impact in quality of life and could improve the clinical course of the disease. Currently, there are no specific recommendations for nutritional supplementation in HF patients, generally it is accepted that it should be started when oral intake is insufficient. Typically, hypercaloric ONS (1.5–2 kcal/mL) are recommended, and if necessary, high-protein, as they appear to improve inflammatory status, quality of life, and survival in patients with heart failure (HF) IC [27]. There is currently no evidence supporting the use of specific ONS formulations in this population. β-hydroxy-β-methylbutyrate (HMB) has been associated with improvements in nutritional status and a significant reduction in mortality risk compared to placebo in hospitalized patients with cardiovascular and pulmonary events, such as congestive HF [28]. Furthermore, guidelines from the European Society of Cardiology (ESC), the American College of Cardiology (ACC), and the American Heart Association (AHA) recommend considering supplementation with omega-3 polyunsaturated fatty acids, given their demonstrated ability to reduce hospitalization and mortality risk in patients with HF [29]. For instance, omega-3 fatty acid supplementation has been shown to reduce circulating cytokines, not only in HF patients, but also in other clinical scenarios [16, 30, 31]. However, there remains no consensus regarding the optimal dosage and mode of administration.

In this cohort, we observed a significant difference between the incidence of malnutrition according to the GLIM criteria and the presence of sarcopenia, this last can also vary depending on the used diagnosis criteria [32]. Due to significant changes in body weight due to extracellular water content in these patients, functional tests that reflect sarcopenia seem to be more reliable in these patients [33–35]. In line with this, changes in inflammasome components were more evident in patients with sarcopenia, than in patients with malnutrition, furthermore, in patients with malnutrition, only changes in cytokines were observed (CCL2 and CXCL8), while dysregulation in other components of the inflammasome cascade was observed in patients with sarcopenia. These findings are in line with the hypothesis that chronic inflammation is related with HF but it is not the only determinant, other factors, such as sarcopenia, could alter this status for enhancing or deprive the clinical evolution of the disease, even the effect of specific HF-treatment.

Previous publications have described changes in the expression of some inflammasome components in HF. Specifically, NLRP1 is increased in patients with atherosclerosis [36], also in patients with peripheric arterial disease, increased serum levels of triglycerides and very low-density lipoprotein cholesterol [37]. For these reasons, NLRP1 inhibition is considered a putative target for preventing heart-related complications [38]. In our cohort NLRP1 was increased in patients with sarcopenia, and interestingly, the decrease in the expression levels of this component occurred in patients undergoing Mediterranean diet but not in those who received additional nutritional supplementation. In line with this, anti-inflammatory properties of the Mediterranean diet have been previously described [6]. Probably the NLRP3 is the most extensively NOD-like receptor studied in HF context, since it has been associated with myocardial hypertrophy under pressure overload [12]; however, although NLRP3 was expressed in the evaluated patients, it was not overexpressed in patients with sarcopenia and we did not observe changes in its molecular expression after nutritional support.

NLRC4 has been reported to be increased in patients with primary atherosclerosis lesions compared with healthy controls [39], furthermore, a genome-wide association study showed that the NLRC4 inflammasome exert a significant role for IL-18 production in acute coronary syndrome patients, thus promoting the formation of atherosclerotic plaque [40]. The finding of increased levels in patients with sarcopenia, suggests a link between decreased muscle mass and increased cardiovascular risk.

Other inflammasome receptors, such as NLRP12, have been implicated in cardiovascular diseases. Polymorphisms in NLRP12 are associated with increased cardiovascular risk in asymptomatic individuals [41]. NLRP12 expression is elevated in heart failure (HF) patients, with similar increases observed for NLRP1 and NLRP6 [42]. In our study, expression levels of these receptors decreased in the intervention group. Notably, n-3 PUFAs are known to reduce NLRP3 expression in cardiovascular disease [43]; whether they similarly modulate NLRP12 and NLRP6 warrants further investigation, especially when included as part of nutritional interventions.

Analysis of inflammasome activation components revealed upregulation of certain genes, particularly caspases, in patients with sarcopenia. Caspases have been linked to pyroptosis-related sarcopenia [44]. In HF, AIM2 expression has been previously reported regardless of etiology, indicating its potential role in cardiovascular pathology [45, 46]. Similarly, CASP5, upregulated in HF patients with sarcopenia in our cohort, primarily promotes pro-inflammatory signaling and pyroptosis [47], though its specific role in HF remains unclear. Importantly, the expression of AIM2 and CASP5 decreased in the intervention group, highlighting the potential of nutritional support to modulate mechanisms underlying sarcopenia.

We observed a decrease in TLR2 and TLR9 expression in patients receiving ONS. Both genes are involved in cardiomyocyte functional changes; TLR9, activated under pressure overload, promotes inflammation and HF. Studies indicate TLR9 is crucial for inflammatory cell recruitment and cytokine production during cardiac remodeling [48]. Its inhibition prevents and slows left ventricular dilation and dysfunction in vivo [49] and deficiency reduces cardiac inflammation while preserving function [50]. During remodeling, inflammatory activation induces hypertrophy and fibrosis, with isoproterenol stimulating TLR2 expression in mouse hearts, TLR2 knockdown diminishes isoproterenol-induced inflammation and remodeling, suggesting it as a therapeutic target [51]. The downregulation of TLR2 and TLR9 in the intervention group implies that the used n-3 and n-6 enriched ONS may protect against adverse cardiac remodeling in HF patients.

Significant molecular changes were also observed in cytokine pathways. Notably, CHUCK encodes a component of a cytokine-activated complex that inhibits NF-κB signaling, which is vital for cardiac regeneration, apoptosis, and fibrosis. NF-κB influences matrix metalloproteinases and fibrotic mediators, regulating cardiomyocyte activity and the balance between apoptosis and pyroptosis [52]. Additionally, NF-κB is upregulated in patients with peripheric arterial disease and dyslipemia [38]. Our data showed decreased NF-κB and increased CHUCK expression in patients receiving nutritional support plus a Mediterranean diet, reflecting that those patients that received additional nutritional support exhibited increased regulation in this pathway, thus they could benefit of preventing heart fibrosis. Furthermore, in our cohort MAPK14 was increased in patients with sarcopenia, which is in line with results of some in vivo studies that suggest that MAPK could positively regulate muscle atrophy [53]. These findings are clinically relevant since they suggest the molecular basis of the clinical improvement that was observed in patients that received ONS.

Continuing with the analysis of cytokines and apoptosis related components, previous research indicates that nutritional interventions, particularly n-3 PUFAs, can modulate IL-6 levels in cancer patients [30], as well as in middle-aged and older adults [54]. SIRT1, involved in transcriptional regulation, energy metabolism, cell survival, DNA repair, and inflammation [26], modulates myocardial remodeling by affecting oxidative stress, apoptosis, autophagy, and inflammation. Prior studies suggest SIRT1 may protect against HF by inhibiting remodeling [26]. Consistent with other cytokines, SIRT1 expression was significantly reduced in the intervention group, indicating a potential protective effect of nutritional support.

Regarding DNA-damage regulators, CDKN2D is a protein that prevents the activation of CDK kinases; thus, it works as a cell growth regulator that specifically controls cell cycle G1 progression. Its expression in HF has not been described yet, but in other cell types, the negative regulation of this factor suppresses cell proliferation, suggesting that its inhibition would also inhibit heart remodeling, which is related to impaired heart function and clinical evolution [36]. In this cohort, the inhibition of this factor was not correlated with changes in LVEF, but histological mechanisms were not evaluated and the effects of long-term interventions should be explored, since changes may require time to produce relevant clinical changes. Remarkably, we did not observe changes in the only factor that was upregulated in patients with sarcopenia (CDKN1B), reflecting that nutritional support does not affect the molecular expression of this cyclin-dependent kinase inhibitor.

This study has certain limitations, notably the relatively small sample size, which may limit the generalizability of the findings. More importantly, we cannot establish a direct causal relationship between a specific component of the ONS (for example, EPA- and DHA-enrichment) and the observed clinical effects, as other components such as carbohydrates and proteins could also have contributed to the outcomes [55]. HF is also characterized by chronic inflammation, thus, it is not possible to specifically determine the effect of the molecular expression of inflammasome components in sarcopenia or HF. This trial involved the use of a high-protein, hypercaloric ONS, which may have contributed to the observed clinical benefits.For instance, a randomized, placebo-controlled trial published in 2021 showed that whey protein supplementation improved systemic microvascular function in patients with heart failure (HF), enhancing endothelial and microvascular reactivity after 12 weeks [56]. Similarly, a 2023 trial demonstrated that whey protein isolate increased skeletal muscle mass and strength while reducing fat mass, thereby improving physical performance and metabolic profiles in HF [57]. Moreover, a 2024 systematic review of dietary and supplemental interventions reported that individualized nutritional support, including whey protein, thiamine, D-ribose, and L-arginine, can reduce mortality and improve ejection fraction, endothelial function, and quality of life (QoL) in patients with chronic HF at high nutritional risk [58]. For ethical reasons, a placebo arm was not included in this study. Nevertheless, comparisons with other ONS or a crossover design could provide further valuable insights. Considering the clinical benefits observed, additional and more comprehensive clinical trials are warranted to confirm and clarify the specific effects of these ONS. Nonetheless, it is clear that nutritional support represents a promising adjunctive strategy in the management of patients with HF, offering clinical benefits beyond those provided by cardiovascular medications and heart rehabilitation programs. In contrast, this study also possesses several strengths, including its duration (which contrasts to the majority of studies that evaluate nutritional interventions) and the high adherence to the ONS. To the best of our knowledge, this is the first report demonstrating alterations in the expression profile of key inflammasome components in patients with HF in response to a targeted nutritional intervention.

## Conclusions

Taken together, our results reveal that Mediterranean diet in combination with nutritional support can modulate the expression of critical inflammation-related components in patients with HF. This modulation might be responsible of clinical improvement in heart function and recovery. Specifically, the use of hypercaloric, high-protein EPA- and DHA-enriched ONS represents a nutritional intervention that might affect the clinical evolution of patients with previous admissions due to HF. The use of this complimentary approach to specific treatment could represent a cost-effective strategy for improving clinical results and QoL in these patients.

## Supplementary Information

Below is the link to the electronic supplementary material.

Supplementary Material 1

## References

[1] Iyngkaran P et al (2015) Northern Territory perspectives on heart failure with comorbidities—understanding trial validity and exploring collaborative opportunities to broaden the evidence base. Heart Lung Circ 24(6):536–543 25637942 10.1016/j.hlc.2014.12.007

[2] Mentz RJ, Felker GM (2013) Noncardiac comorbidities and acute heart failure patients. Heart Fail Clin 9(3):359–67, cii 23809421 10.1016/j.hfc.2013.04.003 PMC3906631

[3] Wong CY et al (2011) Trends in comorbidity, disability, and polypharmacy in heart failure. Am J Med 124(2):136–143 21295193 10.1016/j.amjmed.2010.08.017 PMC3237399

[4] Sandesara PB et al (2018) The prognostic significance of diabetes and microvascular complications in patients with heart failure with preserved ejection fraction. Diabetes Care 41(1):150–155 29051160 10.2337/dc17-0755 PMC5741155

[5] Ather S et al (2012) Impact of noncardiac comorbidities on morbidity and mortality in a predominantly male population with heart failure and preserved versus reduced ejection fraction. J Am Coll Cardiol 59(11):998–1005 22402071 10.1016/j.jacc.2011.11.040 PMC4687406

[6] Herrera-Martínez AD et al (2024) Nutritional support reduces circulating cytokines in patients with heart failure. Nutrients. 10.3390/nu16111637 38892570 10.3390/nu16111637 PMC11174422

[7] Castillo EC et al (2020) What is the role of the inflammation in the pathogenesis of heart failure? Curr Cardiol Rep 22(11):139 32910299 10.1007/s11886-020-01382-2 PMC7481763

[8] Zheng D, Liwinski T, Elinav E (2020) Inflammasome activation and regulation: toward a better understanding of complex mechanisms. Cell Discovery 6(1):36 32550001 10.1038/s41421-020-0167-x PMC7280307

[9] Latz E, Xiao TS, Stutz A (2013) Activation and regulation of the inflammasomes. Nat Rev Immunol 13(6):397–411 23702978 10.1038/nri3452 PMC3807999

[10] Herrera-Martínez AD et al (2023) Bariatric surgery and calcifediol treatment, Gordian knot of severe-obesity-related comorbidities treatment. Front Endocrinol (Lausanne) 14:1243906 37867510 10.3389/fendo.2023.1243906 PMC10588639

[11] Herrera-Martinez AD et al (2022) Inflammasomes: cause or consequence of obesity-associated comorbidities in humans. Obesity 30(12):2351–2362 36415999 10.1002/oby.23581

[12] Tang X et al (2020) SNO-MLP (S-nitrosylation of muscle LIM protein) facilitates myocardial hypertrophy through TLR3 (toll-like receptor 3)-mediated RIP3 (receptor-interacting protein kinase 3) and NLRP3 (NOD-Like receptor pyrin domain containing 3) inflammasome activation. Circulation 141(12):984–1000 31902237 10.1161/CIRCULATIONAHA.119.042336

[13] Pinar AA et al (2020) Targeting the NLRP3 inflammasome to treat cardiovascular fibrosis. Pharmacol Ther 209:107511 32097669 10.1016/j.pharmthera.2020.107511

[14] Zeng C et al (2020) NLRP3 inflammasome-mediated pyroptosis contributes to the pathogenesis of non-ischemic dilated cardiomyopathy. Redox Biol 34:101523 32273259 10.1016/j.redox.2020.101523 PMC7327979

[15] Herrera-Martínez AD et al (2023) Mediterranean diet, vitamin D, and hypercaloric, hyperproteic oral supplements for treating sarcopenia in patients with heart failure-a randomized clinical trial. Nutrients. 10.3390/nu16010110 38201939 10.3390/nu16010110 PMC10781070

[16] Prokopidis K et al (2023) Does omega-3 supplementation improve the inflammatory profile of patients with heart failure? A systematic review and meta-analysis. Heart Fail Rev 28(6):1417–1425 37340115 10.1007/s10741-023-10327-0 PMC10575807

[17] Herrero-Aguayo V et al (2021) Dysregulation of components of the inflammasome machinery after bariatric surgery: novel targets for a chronic disease. J Clin Endocrinol Metab. 10.1210/clinem/dgab586 34363480 10.1210/clinem/dgab586

[18] Cederholm T et al (2019) GLIM criteria for the diagnosis of malnutrition—a consensus report from the global clinical nutrition community. J Cachexia Sarcopenia Muscle 10(1):207–217 30920778 10.1002/jcsm.12383 PMC6438340

[19] Cruz-Jentoft AJ et al (2019) Sarcopenia: revised European consensus on definition and diagnosis. Age Ageing 48(1):16–31 30312372 10.1093/ageing/afy169 PMC6322506

[20] Gahete MD et al (2018) Changes in splicing machinery components influence, precede, and early predict the development of type 2 diabetes: from the CORDIOPREV study. EBioMedicine 37:356–365 30446432 10.1016/j.ebiom.2018.10.056 PMC6286259

[21] Jimenez-Vacas JMEA (2019) Dysregulation of the splicing machinery is directly associated to aggressiveness of prostate cancer. EBioMedicine. In Press 10.1016/j.ebiom.2019.11.008 PMC7000340 31902674

[22] Vandesompele J et al (2002) Accurate normalization of real-time quantitative RT-PCR data by geometric averaging of multiple internal control genes. Genome Biol 3(7):RESEARCH0034 12184808 10.1186/gb-2002-3-7-research0034 PMC126239

[23] Murphy SP et al (2020) Inflammation in heart failure: JACC state-of-the-art review. J Am Coll Cardiol 75(11):1324–1340 32192660 10.1016/j.jacc.2020.01.014

[24] Deswal A et al (2001) Cytokines and cytokine receptors in advanced heart failure: an analysis of the cytokine database from the Vesnarinone trial (VEST). Circulation 103(16):2055–2059 11319194 10.1161/01.cir.103.16.2055

[25] Rauchhaus M et al (2000) Plasma cytokine parameters and mortality in patients with chronic heart failure. Circulation 102(25):3060–3067 11120695 10.1161/01.cir.102.25.3060

[26] Wang Y et al (2023) Activation of the sirtuin silent information regulator 1 pathway inhibits pathological myocardial remodeling. Front Pharmacol 14:1111320 36843938 10.3389/fphar.2023.1111320 PMC9950519

[27] von Haehling S et al (2017) Muscle wasting and cachexia in heart failure: mechanisms and therapies. Nat Rev Cardiol 14(6):323–341 28436486 10.1038/nrcardio.2017.51

[28] Deutz NE et al (2016) Readmission and mortality in malnourished, older, hospitalized adults treated with a specialized oral nutritional supplement: a randomized clinical trial. Clin Nutr 35(1):18–26 26797412 10.1016/j.clnu.2015.12.010

[29] Oppedisano F et al (2021) PUFA supplementation and heart failure: effects on fibrosis and cardiac remodeling. Nutrients. 10.3390/nu13092965 34578843 10.3390/nu13092965 PMC8471017

[30] Guo Y et al (2023) 3 PUFA can reduce IL-6 and TNF levels in patients with cancer. Br J Nutr 129(1):54–65 35249562 10.1017/S0007114522000575

[31] Hardman WE (2004) (n-3) fatty acids and cancer therapy. J Nutr 134(12 Suppl):3427S-3430S 15570049 10.1093/jn/134.12.3427S

[32] Zhang Y et al (2021) Sarcopenia in heart failure: a systematic review and meta-analysis. ESC Heart Fail 8(2):1007–1017 33576177 10.1002/ehf2.13255 PMC8006658

[33] Benitez-Velasco A et al (2024) Differences in the evaluation of malnutrition and body composition using bioelectrical impedance analysis, nutritional ultrasound, and dual-energy X-ray absorptiometry in patients with heart failure. Nutrients. 10.3390/nu16101535 38794773 10.3390/nu16101535 PMC11124170

[34] Fernandez-Pombo A et al (2021) Relevance of nutritional assessment and treatment to counteract cardiac cachexia and sarcopenia in chronic heart failure. Clin Nutr 40(9):5141–5155 34461588 10.1016/j.clnu.2021.07.027

[35] Bonilla-Palomas JL et al (2016) Nutritional intervention in malnourished hospitalized patients with heart failure. Arch Med Res 47(7):535–540 28262195 10.1016/j.arcmed.2016.11.005

[36] Ceruti JM et al (2005) Induction of p19INK4d in response to ultraviolet light improves DNA repair and confers resistance to apoptosis in neuroblastoma cells. Oncogene 24(25):4065–4080 15750620 10.1038/sj.onc.1208570

[37] Bleda S et al (2016) Elevated levels of triglycerides and vldl-cholesterol provoke activation of nlrp1 inflammasome in endothelial cells. Int J Cardiol 220:52–55 27372042 10.1016/j.ijcard.2016.06.193

[38] Liao Y, Liu K, Zhu L (2022) Emerging roles of inflammasomes in cardiovascular diseases. Front Immunol 13:834289 35464402 10.3389/fimmu.2022.834289 PMC9021369

[39] Borborema MEA et al (2020) Inflammasome activation by NLRP1 and NLRC4 in patients with coronary stenosis. Immunobiology 225(3):151940 32276737 10.1016/j.imbio.2020.151940

[40] Johansson Å et al (2015) NLRC4 inflammasome is an important regulator of interleukin-18 levels in patients with acute coronary syndromes: genome-wide association study in the PLATelet inhibition and patient outcomes trial (PLATO). Circ Cardiovasc Genet 8(3):498–506 25747584 10.1161/CIRCGENETICS.114.000724

[41] Akosile W et al (2018) The inflammasome NLRP12 is associated with both depression and coronary artery disease in Vietnam veterans. Psychiatry Res 270:775–779 30551324 10.1016/j.psychres.2018.10.051

[42] Olsen MB et al (2022) Targeting the inflammasome in cardiovascular disease. JACC Basic Transl Sci 7(1):84–98 35128212 10.1016/j.jacbts.2021.08.006 PMC8807732

[43] Lee KR, Midgette Y, Shah R (2019) Fish oil derived omega 3 fatty acids suppress adipose NLRP3 inflammasome signaling in human obesity. J Endocr Soc 3(3):504–515 30788452 10.1210/js.2018-00220 PMC6371080

[44] Wu J et al (2023) TNF-α contributes to sarcopenia through caspase-8/caspase-3/GSDME-mediated pyroptosis. Cell Death Discov 9(1):76 36823174 10.1038/s41420-023-01365-6 PMC9950087

[45] Onódi Z et al (2021) AIM2-driven inflammasome activation in heart failure. Cardiovasc Res 117(13):2639–2651 34117866 10.1093/cvr/cvab202

[46] Du L et al (2022) The AIM2 inflammasome: a novel biomarker and target in cardiovascular disease. Pharmacol Res 186:106533 36332811 10.1016/j.phrs.2022.106533

[47] Newton K et al (2024) Cell death. Cell 187(2):235–256 38242081 10.1016/j.cell.2023.11.044

[48] Mann DL (2002) Inflammatory mediators and the failing heart: past, present, and the foreseeable future. Circ Res 91(11):988–998 12456484 10.1161/01.res.0000043825.01705.1b

[49] Ueda H et al (2019) Administration of a TLR9 inhibitor attenuates the development and progression of heart failure in mice. JACC Basic Transl Sci 4(3):348–363 31312759 10.1016/j.jacbts.2019.01.002 PMC6610159

[50] Lohner R et al (2013) Toll-like receptor 9 promotes cardiac inflammation and heart failure during polymicrobial sepsis. Mediators Inflamm 2013:261049 23935245 10.1155/2013/261049 PMC3713595

[51] Qian J et al (2023) Toll-like receptor-2 in cardiomyocytes and macrophages mediates isoproterenol-induced cardiac inflammation and remodeling. FASEB J 37(2):e22740 36583707 10.1096/fj.202201345R

[52] Ghiasi M (2024) Investigating the NF-κB signaling pathway in heart failure: exploring potential therapeutic approaches. Heliyon 10(23):e40812 39717608 10.1016/j.heliyon.2024.e40812 PMC11664283

[53] Yuasa K et al (2018) Targeted ablation of p38α MAPK suppresses denervation-induced muscle atrophy. Sci Rep 8(1):9037 29899565 10.1038/s41598-018-26632-w PMC5998077

[54] Kiecolt-Glaser JK et al (2012) Omega-3 supplementation lowers inflammation in healthy middle-aged and older adults: a randomized controlled trial. Brain Behav Immun 26(6):988–995 22640930 10.1016/j.bbi.2012.05.011 PMC3398219

[55] Herrera-Martínez AD et al (2023) Standard hypercaloric, hyperproteic vs. leucine-enriched oral supplements in patients with cancer-induced sarcopenia, a randomized clinical trial. Nutrients. 10.3390/nu15122726 37375630 10.3390/nu15122726 PMC10302788

[56] Lorenzo A et al (2021) Dietary supplementation with whey protein improves systemic microvascular function in heart failure patients: a pilot study. Braz J Med Biol Res 54(6):e10577 33886810 10.1590/1414-431X202010577 PMC8055180

[57] Dos Santos EM et al (2023) Effects of whey protein isolate on body composition, muscle mass, and strength of chronic heart failure patients: a randomized clinical trial. Nutrients. 10.3390/nu15102320 37242203 10.3390/nu15102320 PMC10223081

[58] Yu X, Chen Q, Lou IX (2024) Dietary strategies and nutritional supplements in the management of heart failure: a systematic review. Front Nutr 11:1428010 39464682 10.3389/fnut.2024.1428010 PMC11502353

