# Supplementary material for: Modulation of inflammasome components in patients with heart failure using oral nutritional supplements: investigating the molecular mechanisms beyond the clinical benefit
Source: Eur J Nutr. 2026 Feb 3;65(2):39. doi: 10.1007/s00394-025-03878-5 (PMC12868064; doi:10.1007/s00394-025-03878-5)
Supplement: Supplementary file 1 — Supplementary Material 1 [file 394_2025_3878_MOESM1_ESM.docx]

**Supp. Table 1: Baseline clinical characteristics of the patients. Comparison between groups based on the nutritional intervention**

| **Characteristics** | **Total (n=38)** | **Mediterranean diet (n=19)** | **Mediterranean diet and OS (n=19)** | ***p*** |
| --- | --- | --- | --- | --- |
| Sex (♂/♀) | 71.1% / 28.9% (11/27) | 31.6 / 68.4 (6/13) | 73.7 / 26.3 (14/5) | 0.50 |
| Age (years) | 67.5 (61 – 78) | 72 (64.5 – 80) | 65 (56 – 72) | 0.06 |
| Tobacco exposure (%) |  |  |  | 0.01 |
| No | 57.9 (22/38) | 42.1 (8/19) | 73.7 (14/19) |  |
| Active | 18.4 (7/38) | 15.8 (3/19) | 21.1 (4/19) |  |
| Previous exposure | 23.7 (9/38) | 42.1 (8/19) | 5.3 (1/19) |  |
| Type 2 Diabetes | 42.1 (16/38) | 36.8 (7/19) | 47.4 (9/19) | 0.38 |
| Previous ischaemic cardiomyopathy | 34.2 (13/38) | 36.8 (7/19) | 31.6 (6/19) | 0.50 |
| Ejection fraction (%) | 33 (25 – 49.5) | 40 (32.5 – 54) | 38 (23 – 35) | 0.46 |
| NT-proBNP (pg/mL) | 4225 (2001 – 7289) | 3678 (1966 – 7203) | 4412 (2177 – 7255) | 0.59 |
| Current weight (Kg) | 78 ± (70.3 – 89.5) | 81 (75 – 90) | 76 (70 – 85) | 0.17 |
| Overweight/obesity (%) | 73.7 (28/38) | 57.1 (16/19) | 42.9 (12/19) | 0.14 |
| Mortality (%) | 13.2 (5/38) | 21.1 (4/19) | 5.3 (1/19) | 0.17 |
|  |  |  |  |  |

**Legend:** Categorical data are presented in percentages and the absolute number in brackets

**Supp. Table 2: Specific primers for human transcripts used in this study. The table includes the NCBI accession number, the primer sequence, and the product size.**

| **Gene** | **Accession Number** | **Primer Sequence (Sense)** | **Primer Sequence (Antisense)** | **Product Size (bp)** |
| --- | --- | --- | --- | --- |
| **1) NLR-/NOD-like receptors** | | | | |
| NLRC4 | NM_021209.4 | GAGGTCCCACAACTCGTCA | GTTTTTCAGAGGGTTCTTTCCA | 102 |
| NLRP1 | NM_033004.3 | CTTTGGGGACTGACGATGAC | GCCAGCTACAGGGAAGTGAA | 104 |
| NLRP3 | NM_004895.4 | CTGAAACAGCAGATGGAGAGTG | GCAGCAAACTGGAAAGGAAG | 91 |
| NLRP6 | NM_138329.2 | AGAAGAAGAGCCTGGGGAAG | TGCCTGAAAGAGTGGATGAA | 110 |
| NLRP7 | NM_001127255.1 | TTCTGGTCAACACCTCCTCA | CCTCCATCATCTCAGCCTTT | 111 |
| NLRP12 | NM_001277126.1 | ACCGACCTTTACCTGACCAA | CCATCCCAAATAACCAGAGG | 112 |
| **2) Inflammasome activation components** | | | | |
| AIM2 | NM_004833.2 | AAAAGGTTAATGTCCCGCTGA | ATTGTTCCAAGGGGCTGAGT | 88 |
| ASC | NM_013258.5 | GATGCTCTGTACGGGAAGGT | GGCTGGTGTGAAACTGAAGAG | 105 |
| CASP1 | NM_033292.3 | TGTTCCTGTGATGTGGAGGA | TCTTTCAGTGGTGGGCATCT | 90 |
| CASP4 | NM_001225.4 | GCAGGACAAATGCTTCTTCA | GTGGTCCAGCCTCCATATTC | 88 |
| CASP5 | NM_001136112.2 | TTGCTGCTGCCACCTAATG | CAAGGTTGCTCGTTCTATGGT | 100 |
| CASP2 | NM_032982.4 | AGCTGGCATATAGGTTGCAGTC | ATCCCCTCCAGAGCGAAAT | 104 |
| CASP8 | NM_001400660.1 | GTTCCTGAGCCTGGACTACATT | ACAGATTGCTTTCCTCCAACA | 110 |
| CASP9 | NM_001229.5 | TGGACGACATCTTTGAGCAG | GAAATTAAAGCAACCAGGCATC | 119 |
| GSDMD | NM_024736.7 | GGTGCCTCCACAACTTCCT | TGTCCAAAAGCTCCAGTTCC | 117 |
| IFI16 | NM_001206567.1 | ACTGAAGGAGCAGAGGCAAC | ACTGGGCGTTTTTGGAGA | 98 |
| IL1B | NM_000576.2 | TGGCAATGAGGATGACTTGTT | TGGTGGTCGGAGATTCGT | 120 |
| IL18 | NM_001562.3 | CCAAGGAAATCGGCCTCT | CCATACCTCTAGGCTGGCTATC | 113 |
| MYD88 | NM_001172566.2 | CGCCTGTCTCTGTTCTTGA | GTCCGCTTGTGTCTCCAGTT | 114 |
| P2RX7 | NM_002562.5 | GACACCGCAGACTACACCTTC | GTGGGATACTCGGGACACA | 107 |
| TLR1 | NM_003263.4 | TGCCACCCTACTGTGAACCT | GGTGCTCAACCCCAGAAAT | 117 |
| TLR2 | NM_001318787.2 | CTTCTGGAGCCCATTGAGAA | CCAGGTAGGTCTTGGTGTTCA | 82 |
| TLR4 | NM_138554.4 | CCTTTCCAGCAACAAGATTCA | AAGTTCATAGGGTTCAGGGACA | 108 |
| TLR8 | NM_016610.4 | CCTGGCTCACCATTTGTTTT | TTGGGATGTGGAAAGAGACC | 94 |
| TLR9 | NM_017442.4 | TACCTTGCCTGCCTTCCTAC | AAGTGGGGCACAGACTTCA | 87 |
| **3) cytokines and inflammation/apoptosis related components** | | | | |
| CCL2 | NM_002982.4 | CCCCAGTCACCTGCTGTTAT | AGCTTCTTTGGGACACTTGCT | 101 |
| CCL7 | NM_006273.4 | CCTAAGCAGAGGCTGGAGAG | CTTCTGTGTGGGGTCAGCA | 114 |
| CCR1 | NM_001295.3 | TGAGCAGAGCAGACATTTGG | AGGCGTAGATCACTGGGTTG | 89 |
| CCR5 | NM_000579.4 | GATAGTCATCTTGGGGCTGGT | CCTGTGCCTCTTCTTCTCATTT | 106 |
| CHUCK | NM_001278.4 | CTTGGAGAGATACAGCGAGCA | CAACCTCAGCATAGTGGATGG | 104 |
| CXCL2 | NM_002089.4 | AGCCCCAACCATGCATAA | GAGGAGAGCTGGCAAGGAG | 86 |
| CXCL3 | NM_002090.3 | AACCGAAGTCATAGCCACACTC | GTTGGTGCTCCCCTTGTTC | 109 |
| CXCL8 | NM_000584.4 | CTGTGTGAAGGTGCAGTTTTG | GGTGGAAAGGTTTGGAGTATGT | 82 |
| CXCR1 | NM_000634.3 | AATCTGTCCCTGCCCTTCTT | CCATTTTGCTGTGTCATTTCC | 106 |
| CXCR2 | NM_001557.4 | TGGGCAACAATACAGCAAAC | GGGTGAATCCGTAGCAGAAC | 105 |
| DCR2 | NM_003840.5 | CAAGGAGGAGGAGTGTCCAG | ATAGCAGGCAAGAAGGCAAA | 119 |
| IFNG | NM_000619.3 | TTGGGTTCTCTTGGCTGTTACT | ATCCGCTACATCTGAATGACCT | 96 |
| IL6 | NM_000600.5 | ACCTTCCAAAGATGGCTGAA | TCAAACTCCAAAAGACCAGTGA | 97 |
| IL6R | NM_000565.4 | AAGGCTGTGCTCTTGGTGAG | TGGGACTCCTGGGAATACTG | 83 |
| MAPK14 | NM_001315.2 | TCCAGACCATTTCAGTCCATC | CGTCCAACAGACCAATCACA | 100 |
| NFKB1 | NM_021975.4 | CCTGTCCTTTCTCATCCCATC | TGCCAGAGTTTCGGTTCACT | 85 |
| SIRT1 | NM_012238.5 | TGTTATTGGGTCTTCCCTCAAA | GCAGATGAGGCAAAGGTTCTC | 107 |
| TGFB1 | NM_000660.7 | GCAACAATTCCTGGCGATAC | ACAACTCCGGTGACATCAAAA | 88 |
| **4) Cell-cycle and DNA-damage regulators** | | | | |
| ATM | NM_001351834.2 | GCATTCAGATTCCAAACAAGG | GGCTGATACATTTGGTTTTGCT | 118 |
| CDKN1A | NM_001291549.2 | TGCCCAAGCTCTACCTTCC | CACATGGTCTTCCTCTGCTGT | 116 |
| CDKN1B | NM_004064.4 | ATAAGGAAGCGACCTGCAAC | TTGGGGAACCGTCTGAAA | 88 |
| CDKN2A | NM_000077.4 | GACATCCCCGATTGAAAGAA | CAGTTGTGGCCCTGTAGGA | 91 |
| CDKN2D | NM_079421.3 | AACCGCTTCGGCAAGAC | GCTGGCACCTTGCTTCA | 84 |
| **5) Housekeeping genes** | | | | |
| ACTB | NM_001101 | ACTCTTCCAGCCTTCCTTCCT | CAGTGATCTCCTTCTGCATCCT | 176 |
| GAPDH | NM_002046 | AATCCCATCACCATCTTCCA | AAATGAGCCCCAGCCTTC | 122 |
| HPRT | NM_000194.2 | CTGAGGATTTGGAAAGGGTGT | TAATCCAGCAGGTCAGCAAAG | 157 |
